# ‘The body says it’: the difficulty of measuring and communicating sensations of breathlessness

**DOI:** 10.1136/medhum-2019-011816

**Published:** 2021-01-28

**Authors:** Alice Malpass, Coreen Mcguire, Jane Macnaughton

**Affiliations:** 1 Centre for Academic Primary Care, Population Health Sciences, University of Bristol, Bristol, UK; 2 Department of History, University of Durham, Durham, UK; 3 Department of Anthropology, Durham University, Durham, UK

**Keywords:** medical humanities, respiratory medicine, social anthropology

## Abstract

Breathlessness is a sensation affecting those living with chronic respiratory disease, obesity, heart disease and anxiety disorders. The Multidimensional Dyspnoea Profile is a respiratory questionnaire which attempts to measure the incommunicable different sensory qualities (and emotional responses) of breathlessness. Drawing on sensorial anthropology we take as our object of study the process of turning sensations into symptoms. We consider how shared cultural templates of ‘what counts as a symptom’ evolve, mediate and feed into the process of bodily sensations becoming a symptom. Our contribution to the field of sensorial anthropology, as an interdisciplinary collaboration between history, anthropology and the medical humanities, is to provide a critique of how biomedicine and cultures of clinical research have measured the multidimensional sensorial aspects of breathlessness. Using cognitive interviews of respiratory questionnaires with participants from the Breathe Easy groups in the UK, we give examples of how the wording used to describe sensations is often at odds with the language those living with breathlessness understand or use. They struggle to comprehend and map their bodily experience of sensations associated with breathlessness to the words on the respiratory questionnaire. We reflect on the alignment between cognitive interviewing as a method and anthropology as a disciplinary approach. We argue biomedicine brings with it a set of cultural assumptions about what it means to measure (and know) the sensorial breathless body in the context of the respiratory clinic (clinical research). We suggest the mismatch between the descriptions (and confusion) of those responding to the respiratory questionnaire items and those selecting the vocabularies in designing it may be symptomatic of a type of historical testimonial epistemic injustice, founded on the prioritisation of clinical expertise over expertise by experience.

## Background

### Breathlessness as sensation

Anthropologists have traditionally been interested in exploring the inter-relatedness between people’s lived experiences and the biomedical field as a cultural system. Anthropologists view doctors and biomedical thinking as co-producers of the cultural categories that frame people’s bodily experiences and expressions: ‘Bodily experiences do not take place, nor are they expressed, in a vacuum. Biomedicine is a key actor in defining categories through which we experience and express our bodies’.1 This may be even more so when talking about breathlessness with its ‘incommunicable’ sensorial qualities.2 In short, senses are not precultural but are embodied experiences in culturally recognisable (and prescribed) forms.3

Hinton and colleagues distinguish between monomodal sensations (referring to sensations such as the level of oxygen and carbon dioxide in the blood, provided by receptors in the vascular system) and polymodal sensations (referring to sensations which result from several different sensory modalities).4 Shortness of breath is described as a polymodal sensation as it derives from muscle and tendon tension in the respiratory muscles in the chest and neck, the feeling of skin pressure over the stomach as it fails to rise on the inhale, and gastrointestinal tract distension if the abdomen becomes distended, preventing normal inhalation.

As our object of study is the multimodal sensation of breathlessness (and attempts to describe and express it vs attempts to measure and report it), we have approached the study of breathlessness from a sensorial anthropology vantage point.5 The anthropology of the senses is one of numerous approaches that emerged out of the sensory turn in the humanities and social sciences beginning in the 1990s.6 Laplantine, one of the key theorists of the sensorial nature of ethnography, does not view the senses as objects of study but as ways into understanding and perceiving the multiplicity of lived experience. His first book to be translated into English, *The Life of the Senses*, reminds us in the prologue that our role as anthropologists is to critique Eurocentric ways of seeing—one of which is the hierarchical distinction between knowing and sensing. One of the major contributions of the sensorial turn in the humanities has been to locate the sensorial within a social and cultural process, what Howes describes as ‘the sociality of sensation’ in which the perceptual is political—not private and subjective the way psychology would have it.7 As Laplantine previously observed, ‘there exists a political and a historical dimension to sensory experience, which exceeds what individuals can consciously experience’.8

The growing recognition that the sensorium is a social formation is relevant then to the endeavour in this article: to understand the potential for a mismatch to occur between the language used by those living with breathlessness to describe their experience of this multimodal sensation and the language used by biomedicine in its attempts to know, measure and quantify that multimodal sensorial experience. Hinton, Howes and Kirmayer list oxygen and carbon dioxide as among the 11 modalities of sensory experience. Yet despite breathing (unlike say kidney function, cholesterol or blood pressure) being *both a measurable physiological process and an experience we can have insight into via our senses*, in their seminal article on the definitions and research agenda for an anthropology of the senses it is clear that anthropology has paid such little attention to the sensations of breathing and breathlessness.9 Recent exceptions are Megan Wainwright’s ethnographic work in Uruguay exploring how the sensation of breath and breathlessness is culturally situated in local perceptions of air outside the body10; and Brian Lande’s work which explores how inhabiting an institution (such as the army) means ‘learning to breathe in culturally distinct ways’.11 Even this work is focused less on the sensation and more on how breathlessness is culturally mediated. Wainwright’s other work in Uruguay asked participants to draw their lungs: ‘If we could look inside your chest now, what would you see?’12 Her work found participants’ imagininings of their lungs (and its reduced capacity) were shaped by the medical images that form part of everyday clinic visits and pulmonary rehabilitation. Wainwright concludes from this that medical technology and images impact participants’ embodiment. Her work exemplifies how biomedicine is a key actor in not only defining the categories through which we experience and express our bodies, but the way we imagine and embody our physical and sensorial forms.

### The measurement of breathlessness

Until recently, most clinical measurements of dyspnoea, the clinical term for breathlessness, treated it as a single entity. Within the biomedical domain breathlessness is discussed as resulting from a complex interaction of physiological, psychosocial, social and environmental factors. The authors of a recent multidimensional model for dyspnoea (MDP) suggest that breathlessness, like pain, comprises multiple components that can be measured as different entities.13 The MDP separately measures the immediate unpleasantness or discomfort of breathing (A1 domain), presence and intensity of five sensory qualities, and intensity of five emotional responses of breathlessness. The MDP is innovative because it takes into account the importance of emotion as a consequence of breathlessness and one that significantly influences how people respond to it.14 There are a plethora of assessment tools available to measure breathlessness; a review in 2007 identified 33 measures, concluding that there is no single instrument that encompasses all the components of the sensation of breathlessness.15 We deliberately chose the MDP (created after the date of the 2007 review) for its innovative measurement of breathlessness as a multidimensional phenomena. During the period of our study, it was just being published as the latest contribution to the field of measuring breathlessness experience and perception of its sensory qualities. It has since been translated and used in several languages, including French (language-specific versions for France, Belgium and Canada), German and Dutch (language-specific versions for Belgium and the Netherlands), English (language-specific versions for Canada and the UK), and Swedish.16, 17 Validation studies in outpatients have been performed in Australia and France and in a Portuguese version, but not yet in the UK.18, 19 The MDP, as a new measure of breathlessness, is not yet validated in the UK, is not routinely used within clinical settings, and due to its length is most likely to be used in respiratory-based research rather than in clinical practice.20 It was also of interest to us because of claims that MDP is sensitive in detecting *changes in dyspnoea sensation* evoked by different physiological stimuli.21

The primary focus of most studies of breathlessness sensation has been to determine whether different patterns of sensory qualities discriminate among various diagnoses, with more recent studies exploring the extent to which sensory qualities of breathlessness may vary with changes in health status within a single diagnosis.22 The authors of the MDP assure us that the ‘Content validity of the MDP items is strong because each item is based on earlier studies in which clinical experts and patients evaluated their clarity and salience’.14 Two of these foundational studies assessed the validity of the investigator’s descriptors of breathlessness by consulting a panel of experts.23, 24 Parshall’s work is distinct in that prior to administering the descriptor breathlessness questionnaire, researchers asked one open-ended question: ‘what words would you use to describe what your breathing felt like when you decided to come to the emergency room?’25 Parshall then evaluated the similarity to open-ended characteristics with the number and percentage of descriptor choices. The claims for a strong content validity of the MDP questionnaire items have also been strengthened by a recent independent comparison of the MDP with other measures of breathlessness reporting that the MDP ‘was readily understood by patients’.26

The MDP attempts to measure the ‘incommunicable’ different sensory qualities (and emotional responses) of breathlessness. Its authors tell us the MDP was ‘developed from existing instruments for pain and dyspnea and subsequently refined through laboratory work’.27 It comprises 12 items: an immediate sensory intensity item, an immediate unpleasantness item, five items addressing sensory qualities (eg, tightness, muscle work) and five emotional response items (eg, frustration, anxiety). Sensory qualities of dyspnoea were reduced from a list of 19 descriptors to 5 descriptors group based on previous factor analysis in patients and laboratory use in healthy subjects and patients. The emotional response items were adapted from pain research.

### Critiques of quantitative measurement

A medical humanities critique of the MDP and how it has been compiled identifies a number of problems that are not acknowledged by the authors and which derive very much from a clinical culture that does not always recognise the limitations that culture imposes. First, the basis they use to connect people’s ‘incommunicable’ sensory experience to physiological mechanisms is by offering word descriptors of breathlessness sensations to subjects and asking them to choose the best fit, rather than allowing a range of possible descriptors to emerge unbidden from their respondents, as might happen in a qualitative research context. This method is likely to be highly suggestible to patients and may also lead to a narrow range of descriptors, making it easier to ally them to discrete mechanisms. Most early work relies on consulting a panel of experts to check the content validity of descriptors. When qualitative descriptors of breathlessness *are* elicited in these early studies, the sample is ethnically homogeneous. For example, only 2 of the 104 patients included in Parshall’s study in 2002 were non-white.28 This leads on to our second point: the language used by different ethnic groups may differ even when apparently describing the same sensation.29 The physiologists admit that they use people from very similar groups in their experiments, such as white US male college students. Finally, what is most striking about Lansing’s model is that emotional response is described as deriving *from* the sensation of breathlessness. It is this assumption that we will begin to dismantle in this article, suggesting instead that people’s experience of their chronic breathlessness profoundly colours *how the sensation is perceived*. The problem with the laboratory-based approach is that this experimental work is largely carried out on normal subjects whose bodies and minds have not been subjected to years of chronic breathlessness and the effects that may have on physiology and neural mechanisms. Scholars such as Steven Epstein and Heather Prescott have drawn attention to the data gap and resulting health disparities occasioned by research studies using only the average white male as the normative standard.30–32 However, few scholars have explored the extent to which using data only related to the normal body results in a ‘disability data gap’. Yet studies using real patients are a challenge for people whose condition does not enable them to spend time lying flat in the enclosed tunnel of an MRI scanner.33 Our neuroscience collaborator, Kyle Pattinson and colleagues, acknowledge this when they write:

Replicating the emotional component of dyspnea in a laboratory environment is difficult as laboratory dyspnea does not cause the existential fears dyspnea sufferers encounter in daily life, hence patient studies will be necessary in order to fully comprehend all aspects of dyspnea.34

Those at the forefront of laboratory work, such as Lansing and colleagues, are aware of the differences between laboratory and patient dyspnoea, admitting that ‘It is likely that patients studied in the controlled atmosphere of the laboratory and given assurances of the safety and limited duration of stimuli will have less emotional response than a patient in a more uncertain life situation’.35

Yet the ‘disability data gap’ continues to be researched and reproduced. There is a need for a critical medical humanities and critical anthropology approach that identifies problems with the scientific methodology and aims to work in a way that brings interdisciplinary insights about experience into dialogue with clinical science to help close the gap between clinical measurement of symptoms and lived experience of sensations. A critical medical humanities perspective suggests instead that the experience of breathlessness is profoundly coloured by prior experience, beliefs and cultural influences. Affect is not just a response to breathlessness but also determines what that experience is like.36

Our aim in this paper, and contribution to this field of sensorial knowledge within anthropology, is to provide a critique of how biomedicine and cultures of clinical research have measured the multidimensional sensorial aspects of breathlessness. In this paper we draw on research using cognitive interviews of respiratory questionnaires and we give examples of how the wording used to describe sensations is often at odds with the language those living with breathlessness understand or use. Those living with a respiratory disease struggle to comprehend and map their bodily experience of sensations associated with breathlessness to the words used to describe symptoms of breathlessness on the respiratory questionnaire.

We argue biomedicine brings with it a set of cultural assumptions about what it means to measure (and know) the sensorial breathless body in the context of the respiratory clinic (or clinical research). We suggest the mismatch between the descriptions (and confusion) of those responding to the respiratory questionnaire items and those selecting the vocabularies in designing it is symptomatic of a type of testimonial epistemic injustice, founded on the prioritisation of clinical expertise over expertise by experience, as well as the prioritisation of the physiological. Broadly speaking, epistemic injustice connotes the doubt that is placed on certain (discriminated) groups’ claims to knowledge.37 In the arena of healthcare, testimonial epistemic injustice affects individuals’ access to treatment as testimonies about their own bodies and health are placed under extra and unnecessary scrutiny.38 As well as the distinctive kind of epistemic injustice affecting disabled people, clinical measurement technologies can compound injustice by introducing biases that have become part of objective assessment. Simultaneously, hermeneutical injustice can impact on groups whose experiences are not reflected in biomedical terminology. Consequently, dissonance between the aims of the researcher and what participants consider relevant questions to be asked can impact on the validity of questionnaire results. For example, in 1950 the Medical Research Unit for Pneumoconiosis in South Wales visited ‘housewives in the mining valleys’ in Wattstown with a questionnaire designed to explore the impact of economics and the environment on the incidence of pneumoconiosis. However, the relationship of the questionnaire to the measurement of standard of living was not explained to participants, with the result that the researchers caused great offence to the Welsh housewives when they asked personal questions about their husbands’ earnings and how much they spent on clothes and sweets, which were interpreted by the housewives as rude and intrusive. This led to a ‘wives ban’ of the questionnaire, in which many refused to answer any questions. One respondent was moved to write to the local paper, questioning the legitimacy of the questionnaire and arguing:

I agree some of the questions are logical. It is the more personal ones that housewives have taken objections to. Many wives have refused to have anything at all to do with this “inquisition.” Many more have openly admitted that they have given false information, because they will not disclose their husbands’ actual earnings, their personal housekeeping allowance, what they pay for clothing, clubs, insurance’s [sic] etc. etc.39

This historical example highlights how the quest for meaning and the quest for measurement are often at odds when participants and researchers hold conflicting views over the meaning and relevance of the questions posed. We frame our discussion of the MDP by combining a history of science account of epistemic injustice (for those claiming reduced lung function as a result of occupation) with the emerging field of an anthropology of the senses and what it can offer an understanding of ‘symptom experiences’ in relation to breathlessness.

This paper is based on a collaborative work on the Life of Breath project,40 which took as one of its major themes the exploration of ‘symptom discordance’ in relation to breathlessness.41 This means that measured breathlessness in the clinic does not always equate with the patient’s experience. However, our approach wishes to avoid simplistic critiques of the clinical, and rather to find ways of reconciling and benefiting from both perspectives.

## Methods

### The research setting

As a collaboration between three scholars from three disciplines (anthropology, history and medical humanities), our methodological approach is novel, combining cognitive interviewing techniques with ethnographic data collection and historical and interpretative reflections.

The Breathe Easy groups were the first point of face-to-face contact for the research. While receiving no funding from the British Lung Foundation (BLF), the Breathe Easy groups are advertised on the BLF website, with every Breathe Easy group having its own web page with details of when and where the group meets. Local primary care services may also host meetings or support recruiting new members to the group from their patient lists of respiratory patients.

The Breathe Easy groups provide peer support and information for people living with a lung condition and for those who look after them. Within the South West of England there are five Breathe Easy groups, each of whom individually holds regular monthly meetings at community centres. The key focus is for people to meet and talk to others, share their experiences, and learn from each other. At each meeting external speakers are invited to give information and guidance on living with a lung condition and how to cope with the emotional aspects of doing so. There are also social events for those attending. All the participants in this UK-based study would have had access to primary and secondary care services within the National Health Service (NHS), with access to diagnostic and pulmonary rehabilitation—an exercise and education programme designed for people with lung disease who experience symptoms of breathlessness.42 Those who had not been referred to pulmonary rehabilitation via their family doctor would have heard about it through Breathe Easy external speakers (often respiratory clinicians or physiotherapists involved in pulmonary rehabilitation) and/or informal conversations with others in the group. Similar to the racial disparities reported for the uptake of pulmonary rehabilitation, the five Breathe Easy groups visited for this research were attended majoritively, and in most cases exclusively, by white participants.43, 44 The Breathe Easy groups did not reflect the demographics of their local areas and over-represented a particular social group—those who are more likely and willing to access NHS services more easily, having the means to attend groups (ie, their own transport).

### Data collection

Data collection combined attending, participating and observing Breathe Easy group meetings over an 18-month period, which involved various actions of building rapport and a shared sense of reciprocity. Through actions such as bringing and sharing homemade cakes or helping to set up the meeting space and tidy up afterwards, AM discovered opportunities for more informal conversations and observations to take place. AM always remained a guest in these contexts and was never allowed to *serve* tea. In this sense AM did not move beyond the newbie or outsider status in the process of acculturating/socialising herself into the Breathe Easy world. The relationship with individuals deepened through one-to-one interviews which were conducted in participants’ home environments, lasting between 1.5 and 3 hours. Relationships deepened to the extent that it was socially appropriate for AM to bring a housewarming gift when visiting a participant who she knew had recently moved house. Relationships and connections with some key participants remained intact 4 years later, with intermittent email contact, phone call exchanges and requests for information (eg, helping a family friend of one participant prepare for an academic interview), and AM was included in the community experience of losses—being sent the funeral order of service when one participant died. These types of longitudinal connections are indicative of an engaged, reciprocal ethnographic approach.

One-to-one cognitive interviews (described below) were digitally recorded on an encrypted recorder and transcribed verbatim. For the cognitive interviews with 16 participants, a purposeful sampling strategy was adopted to represent (when possible) different stages in the clinical encounter (eg, time since diagnosis, number of exacerbations), as well as sex, age and ethnicity. In line with the cognitive interviewing approach, participants were invited to complete the MDP while ‘thinking aloud’ their thoughts, giving a running account of what was going through their mind as they read the questions and pondered their answer. AM used non-directive, open verbal probing during this process, such as ‘tell me a bit more about what you are thinking?’ and ‘can you say a bit more about that?’ Observation probes were used alongside non-directive probing, such as ‘you’re hesitating, can you tell me why?’, ‘your pen is moving between two options, why?’ and ‘why did you change your answer?’ She followed up with more targeted probes to learn more about the response process, for example by asking ‘what does that term mean to you?’ and ‘why did you choose that answer?’ While the primary method of data collection reported here was cognitive interviewing, AM was also engaged in more sensorial methods of creative data collection45 through collaboration with an arts health practitioner and the development of the ‘letter to the breath’ project, again with the same Breathe Easy groups across the South West. This work and its sensorial methods is reported elsewhere.46, 47 It is mentioned here to contextualise the cognitive interview data reported and emphasise these were not stand-alone encounters, but entangled within a longitudinal and multimodal methodological approach, resembling a critical humanities approach.48

### The alignment of cognitive interviewing with anthropological approach

Anthropology as a discipline is prefaced on the distinction between volunteered information and that obtained through elicitation. In some senses, anthropology was built on a critique of the questionnaire and the survey. Edward Leach, in his work ‘An Anthropologist’s Reflections on a Sociological Survey’, critiqued the *limitations of survey data* and the *ability of ethnographic data to explain inconsistencies:* ‘One indepth micro study can make sense of the most detached survey’.49 Confronted with an extensive survey of land ownership in 57 villages in Ceylon, Leach drew on his fieldwork in just one village to counter the misleading quantitative interpretations. The survey had concluded that a high proportion of villagers were landless peasants. Leach pointed out that many would inherit land from their elders.

Cognitive interviewing takes the distinction between volunteered information and elicited information to a micro level, by comparing survey and questionnaire responses of one individual with a reflective, free-flowing narrative response to the the words and language used by that same participant as they wrestle with each of the individual questionnaire items.

Cognitive interviewing as a method has its origins in psychology rather than anthropology. In the early 1980s, psychologists and survey methodologists deliberately attempted to create a new interdisciplinary field, which became known as CASM–Cognitive Aspects of Survey Methodology. CASM draws on psychological theories of language, comprehension, memory and judgement:

Respondents *first need to interpret the question* to understand what is meant and to determine which information they ought to provide. If the question is an attitude question, they may either retrieve a previously formed attitude judgment from memory, or they may form a judgment on the spot they may need to format their judgment to fit the response alternatives provided as part of the question. Moreover, *respondents may wish to edit their response* before they communicate it, due to *influences of social desirability and situational adequacy. Performance* of each of these tasks is *highly context dependent* and often profoundly shaped by the research instrument.50

But how anthropological is the use of cognitive interviewing? In addition to more recent discussion of the interview as participatory,51 a good place to answer the relationship between anthropology and cognitive interviewing is with the work of Judith Okely.52 Judith was first employed on a research project by a civil servant in the ministry of housing in the early 1970s to gather views of traveller gypsies in England, using a 20-page questionnaire to be delivered to 500 families. Judith describes initially hiding the questionnaires under her bed as any written paperwork was viewed as a signifier of power and authority by traveller gypsies who were at the time mostly illiterate. Judith delegated the job of completing the questionnaire to a social worker and sat back to observe how (in a way similar to the miner’s wives described earlier) ‘gypsies faked or dodged answers to the absurdly intrusive questions. Some said they had never been married before when the researcher knew otherwise…they claimed never to have travelled because they thought this was what the questioner wanted to hear’, leading Judith to privilege the power of observation (instead of self-report assessments) to reveal ‘inconsistencies between what people say they do and what they actually do’.53

### Analysis

AM conducted the analysis for this article. Analysis used both the digital audio file and verbatim transcripts, as the former retains important features needed for interpretative analyses (eg, hesitations, tones of uncertainty, indicators of irritation). Drawing on the framework approach for the practical management of the data and deductive aspects of analysis,54 an Excel grid was created to aid analysis, with each of the MDP items listed as column headings, and within each itemised column two subcolumn headings denoting ‘comprehension’ and ‘answer mapping’ for each item on the MDP. Additional columns summarised data thematically that did not relate directly to the MDP item, such as themes concerned with when participants first noticed they were breathless. Participants were listed in rows. Our approach to analysis was thematic, looking at the language and phrases used by participants in order to identify patterns within and across participants. We examined these themes in relation to the contexts of individuals’ lives and the wider cultural contexts of the Breathe Easy groups. As we were interested in the mismatches between the language used on the questionnaires and the meanings and interpretations of those living with breathlessness, we regard our analysis as interpretative, while at the same time sensitive to the structures and histories influencing the meaning-making process for individuals. In the discussion, we continue this interpretative tone of enquiry into the meaning of our findings.

### Patient and public involvement

The research question was developed alongside patients living with chronic obstructive pulmonary disease (COPD) whose experience suggested that clinicians often fail to enquire about the symptom of breathlessness and that the experience of completing questionnaires in clinical settings does little to meet the concerns and needs about living with breathlessness. Those living with respiratory illness were consulted through regional Breathe Easy groups and public engagement activity. The findings from this article will be made available in a shortened format to chairs of the Breathe Easy groups who were involved in the patient and public involvement consultation process.

## Findings

AM would have been in contact with around 100 participants attending the five Breathe Easy groups during the 18 months of fieldwork. Of those, 16 participants agreed to take part in a cognitive interview at their home, who ranged in age from 69 to 90 years, with an equal number of men and women recruited. Everyone who attended the Breathe Easy groups were white, so recruitment was limited to white adults, all British with the exception of one man who was Italian. The length of time since a breathlessness-related diagnosis ranged from 2 to 17 years, although three participants had experienced breathing discomfort for much longer prior to diagnosis. Some had childhood experiences of breathing discomfort relating to repeat episodes of pneumonia, tuberculosis, childhood asthma or partial removal of the lung as a young adult as a result of a successfully treated tumour. One participant had had repeated episodes of pleurisy prior to her diagnosis, and another had had *Haemophilus influenzae* infection. Types of diagnosis included two participants with asthma, and the rest with variations in COPD such as pulmonary fibrosis, bronchiectasis and emphysema. Of the 16 participants, 3 were housebound (attending Breathe Easy monthly groups being a rare ‘out of the house’ experience), while over half of the sample had had more than one unplanned hospital admission as a result of their breathlessness. Since the data were collected in 2017, two of the participants are known to have died.

For the purpose of this paper, we present the items on the MDP which caused the most difficulty in terms of comprehension and answer mapping (the process by which a participant tries to map their lived experience to the question being asked and the answers available to them on the questionnaire). For each of the four statements we go on to discuss from the questionnaire, participants try to decide whether to tick ‘the statement does apply’ and ‘the statement does not apply’ in relation to feeling breathless when involved in an activity they have already identified at the beginning of the questionnaire (eg, when hanging out washing, when mowing the lawn). The same four statements appear a second time in the questionnaire, with participants invited to rate the intensity of each statement on a scale of 0–10 (with 10 being as intense as I can imagine).

### ‘I am not getting enough air, I am smothering, I am hungry for air’

The triple barrelled nature of this questionnaire item caused answer mapping problems, despite the guidance stating it is okay if just one statement applies; the lack of recognition with the other two statements caused uncertainty in terms of how to answer. Participants also identified that they used pacing to avoid getting to a stage of ‘not getting enough air’. The word ‘air’ instead of ‘breath’ also caused difficulties in answer mapping, leading participants to reflect on personhood and ask ‘who breathes’, the body or the self? Participants separated out the bodily mechanics of the lungs and body wanting to fill up with air from their sense of self, making it problematic to answer this question.

Most participants identified with just one of three statements. The phrase ‘I am not getting enough air’ resonated with their experience of ‘fighting for air’, whereas descriptions of sensations of smothering or feeling hungry for air caused confusion and lack of recognition. The following response is typical:

“I am not getting enough air” makes sense to me. But smothering, I can’t see how I could be smothering. Smothering to me means you’ve got something over your face, over your mouth. No, I don’t feel hungry for air. I just feel short of air. So the question, I don’t really understand the question. (BEBJ)

Smothering was read as literally meaning something had to be over the person’s face to be feeling the sensation, and feeling ‘hungry for air’ was equated with being in an enclosed space, such as underground or under the water diving and running out of oxygen. Both were associated with panting and gasping for breath. These two descriptions had much less resonance for participants in our study.

One participant paced themselves when walking or carrying things in order to avoid the experience of ‘not getting enough air’: “so I stop to get, get a little bit of air in” (BEB9). For another participant they purposively tried not to get too much air because it was painful, yet they longed to be able to breathe deeply. In response to this questionnaire item they explained: “if i get a lung full of air now my chest hurts, why put yourself in pain (I shallow breathe)…you try explaining that on paper, its difficult” (BECR).

The use of the word ‘air’ made sense to those who thought in terms of ‘getting a lung-full’. In contrast, instead of ‘I am not getting enough air’, one participant preferred the phrase ‘I cannot get my breath’ because for her the air is all around her in a non-problematic way and what she struggles to get enough of is her breath:

No, not the air. It’s sort of, it’s all around us, that’s air. (It’s) Not sort of “I can’t get any air”. It’s “I cannot get my breath”…the statement is tricky because it could mean lots of things, I don’t understand the question. (BEBAJ)

The air transmutes into (out of reach) breath only at the point when a person tries to inhale. At what point does air come to be experienced as ‘my breath’, and what are the links between a breathing self and their environment? One participant could not identify with the idea that it *was him* who was ‘hungry’ for air or ‘wanting air’. He felt it was *his body* which was wanting air, leading him to separate out the bodily mechanics of the lungs and body wanting to fill up with air from his sense of self:

my body is craving for air, but I don’t feel that I am craving for air, I’m just dealing with my asthma, that’s what I’m thinking (reads out question again)…the body may be hungry for air *but i’m not*, its not part of my consciousness, my understanding of asthma is that the body kicks in and says “you need more air, you need more air” so its not “I feel *I* need more air, *I’m* smothered” its *the body says it*, it’s part of a process of my asthma, but not a part of a process of me. (BEBAL)

The separation of the body (and its need for air) from a sense of self is something distinct we see in relation to breathing, but not for example in relation to sensations of pain. Finally one participant framed her experience of breathlessness even more in terms of appropriate biological function; she preferred to think of her body needing oxygen rather than air:

but I’m focusing more on the first bit: “I’m not getting enough air”.…But I [clears throat] I don’t think of it as air; I think of it as oxygen, I suppose. Yeah, you know, it’s, it’s oxygen, and the body needs oxygen to, er, work appropriately, yeah. (BEWSM)

### ‘I am breathing a lot’

This term may seem straightforward but it caused a lot of confusion, with many not understanding what it was asking them. Participants interpreted it in various ways; for some it described the opposite experience of feeling breathless, while others assumed it referred to healthy breathers involved in exercise. While some participants interpreted the phrase to mean gasping for breath, hyperventilating or breathing fast, it still did not resonate with them because it could be applied to a healthy breather. To overcome these issues the participants recommend the phrase be altered to ‘I am breathing a lot *with difficulty*’.

Although the word ‘breathing’ instead of ‘getting air’ (as in the previous questionnaire item) made more sense to participants, this statement still caused a lot of difficulty because it was often interpreted to mean the opposite of being breathless:

breathing a lot, no I’m not, I’m struggling to get breath, so no (does not apply) “I don't understand breathing a lot”, I’m not breathing a lot *I’m trying to get what breath there is*…so that does not apply. (BEC12)

Other interpretations of the phrase was that it might refer to a healthy breather who is engaged in aerobic exercise: “it’s not in my experience, breathing a lot sounds like healthy breathing, pushing yourself aerobically, it doesn’t sound relevant to me” (BEC11).

While some understood the phrase to mean gasping for breath, hyperventilating or breathing fast, it still did not resonate because it could be applied to a healthy breather, so if the phrase was altered to ‘I am breathing a lot *with difficulty’* it made more sense:

does that mean breathing fast? Gasping as it were? I’m breathing, you know, I’m breathing with difficulty, I want to cross it out, it does apply, I am struggling, I’m going to put in brackets [on the form] “with difficulty.” (BEC14)

And again like the previous questionnaire item, some participants paced themselves so they never got to the stage of panting or gasping for breath:

do they mean rapidly (laughs)? Well, yeah, I honestly don’t understand that…I breathe normal until I’m going up hills…but I don’t even pant going up the hills because I stop and I take in (air) before it really hurts me. I am putting not apply. (BeBS21)

For those who did not pace themselves when active, the statement made sense in terms of ideas: a fast, racing breath: “when I am walking uphill and I’ve got to do it quickly, I am breathing, I’m breathing a lot. My breathing gets faster and faster and faster” (BAJ).

Only one other participant found the statement straightforward; he understood ‘breathing a lot’ in terms of ‘breathing harder’ and the physical effort needed to pump more air into the lungs. When designing the MDP, an earlier version of this item was accompanied by helper descriptions in parentheses (breathing rapidly, deeply or heavily), but subjects in the laboratory commented that these were confusing—for example someone may be breathing rapidly but not deeply—and so the MDP authors report removing these descriptors, ‘leaving just “breathing a lot” to capture a sense of increased ventilation’.55 Our findings for this item suggest participants rarely understood the phrase ‘I am breathing a lot’ to mean an increase in ventilation. It was problematic because it could apply to normal, healthy aerobic breathlessness as well as pathological breathlessness, which left them feeling their experience was not truly represented by the statement. Interestingly, the work on which the MDP descriptors are based also found the statement ‘I felt I was breathing more’ was confusing for those living with breathlessness. As a result Moy *et al*’s team changed the statement to ‘breathing harder than usual’,56 but Parshall’s team kept the original wording and found less than half his sample chose this descriptor, with many feeling perplexed as to its meaning.57 With such accounts, corroborated by our findings, it is hard to understand why the MDP included this descriptor.

### ‘My breathing requires muscle work or effort’

Participants interpreted this item in various ways; for example effort was taken to mean having to stop an activity ‘to get a bit of breath in’, or the effort needed to stop to control and calm the breathing, or the effort involved in an activity being so challenging that help is required from others, or the additional effort needed to breathe because of changes to the air, such as the steam when showering. There was confusion over which muscles were being referred to—muscles in the whole body or the muscles involved in breathing. There was also a lack of comprehension over what muscles are involved in breathing, with some participants talking about stomach muscles, others unsure what muscles (if any) are involved in breathing, and others describing the lungs as a muscle. Some interpreted muscle effort and work to mean using the hands on the sides of the diaphragm to help the chest exhale, while others thought it might refer to the muscles involved in leaning over (a sink or chest of drawers) when breathless. Some equated muscle effort with the sensation of tightness. The biggest comprehension issue was whether muscle effort involved in breathing is conscious or not.

One participant focuses on trying to comprehend the meaning of ‘muscles’:

Well, I suppose I’ve got to have a little bit of muscle work, haven’t I, to breathe? I’ve got to stop [an activity], and that’s effort. Um, but I don’t know how you do that, not apply—it does apply? Well, you’re bound to have a bit of, er—I mean muscles, what, in your stomach? (BECR)

This item on the MDP caused a lot of comprehension issues for three reasons: either because participants felt unsure which muscles were involved in breathing, or because they distinguished between rest and activity, or because muscle work and effort supposed a conscious awareness, which many felt was not the case either when breathless or when breathing more normally at rest: “Its beyond muscle effort or work, that, you are not conscious, you are just struggling” (BEC14).

Whether breathing was a struggle or not, the effort involved was considered unconscious: “‘My breathing requires muscle work or effort. Well, I’ll put 3, for the simple reason that it must do, but it doesn’t make sense—it’s not something I think about, I just do it” (BeBAM).

The confusion over comprehending the term ‘muscle work’ hangs on two competing interpretations of the questionnaire item: that activity (such as walking or mowing the lawn) involves muscle work and effort and results in breathlessness versus viewing breathing itself involving muscle work and effort. The difficulty comes because at rest breathing is not problematic, so the muscle involved in breathing is viewed as ‘natural’, involving no conscious effort.

For others, while they too distinguished between exertion and walking, the idea that muscular effort was involved in breathing was a straightforward question; although they incorrectly described the lungs as a muscle, they were aware of the increased sensations of muscle work and effort in their chest:

Well, I just feel that my muscle, my lungs, because I suppose your lungs are a muscle, are working a lot. There’s more effort than when I’m just—if I’m just walking along the road I *don’t notice that anything is happening*…But when I go uphill *I can feel them—and I think they’re moving…my chest is moving more*. (BeBAJ)

A lack of medical knowledge about the muscles involved in breathing (diaphragm and intercostal muscles) made it easier to answer the question because she focused on the sensations of her chest moving and compared this with other times when she ‘didn’t notice anything was happening’. For others the confusion came because they assumed the questionnaire item was suggesting the lungs are a muscle:

“when exercising, my breathing requires muscle effort or work”, I don’t, it just [mimics] “gasps gasps”, is that me? my muscles working or my brain telling my lungs to start working, that’s not muscle effort, *I don’t think so, to me my lungs haven’t got any muscles, have they?* your diaphragm, if you breathe in, deeply from your tummy up, that pushes your lungs up *but thats your diaphragm working not your lungs working isn’t it?* (BeCR)

For another participant the idea of muscle effort and work only made sense when seen as related to the sensation of tightness and effort needed to support the exhale:

“Breathing requires muscle work or effort.” Oh right, yes, a bit like tight chest…When [my mother] was wheezy…one thing she would do is, as she breathed out, she would put her hands on the side of her rib cage and push downwards, pushing the rib cage in at the base to help with her breathing out. So that’s muscle work and effort. (BE-CL)

### ‘My breathing requires mental effort or concentration’

Similar to the previous questionnaire item this caused some difficulty in comprehension and was interpreted in a variety of ways. Participants responded more easily to the word ‘concentration’ than ‘mental effort’. Mental effort was interpreted to mean three different types of thinking: the thinking involved in avoiding becoming breathless, the thinking involved in pacing oneself or persevering in an activity that makes one breathless, and the thinking involved in applying certain breathing techniques or the effort it takes to relax or distract oneself during a breathless episode. For others the terms just caused confusion or were interpreted to mean ‘mental health’. Confusion for some was linked to viewing the breath (and breathlessness) as an automatic bodily process, beyond conscious thought. A range of emotions were associated with the phrase ‘mental concentration’, including worry, fear, stoicism, frustration, courage and perseverance. Again comparisons were made between the mental effort involved when at rest compared with when active or when ‘it gets out of hand’. Participants associated the all absorbing nature of being concentrated on their breath with a poor quality of life.

For one participant, mental concentration was interpreted as the breath ‘grabbing [her] attention’ and was associated with perseverance, challenging automatic negative thoughts about the limitations of the body:

Yes, I mean it requires concentration in that it takes, it grabs my attention, and I have to act, just stop a minute before I do the last few. So I don’t know what—how to express that. Oh dear…Well, *it’s more about determining to do it*, thinking, “Oh, I can’t do that,” and then thinking, “You’ve flipping well got to…it’s this [points to head] telling the rest [points to body] what to do”…And getting on and doing it. (BE-WS-D)

Despite the apparent comprehension of the phrase ‘mental concentration’, the participant still is unsure how to answer: “I don’t know how to express that, oh dear.” The phrase ‘mental concentration’ does not seem a comfortable fit with her experience when she is grabbed by breathlessness. Similarly, experiences of concentration were described more in terms of pacing, feeling the need to stop and wondering “when my breathing will get better.” This is more complex and nuanced an experience than the statement ‘mental concentration’. One participant initially struggles to comprehend the meaning of this questionnaire item, viewing breathing as automatic and unconscious:

I don’t find that applies at all…you automatically breathe, so that doesn’t apply, or does it apply?…I’m thinking breathing to me is automatic, its not an effort, I don’t need to concentrate to breathe, it’s automatic…that’s a difficult one. (BE-WS-S)

However when later prompted by the researcher to think of the time when she is breathless (washing in the morning), suddenly the question makes sense:

‘My breathing requires mental effort or concentration’. So, yes it does apply. Because when I’m getting washed I’m getting so out of breath, you’ve got to try then to concentrate on your breathing techniques to get you over your breathlessness. (BE-WS-S)

Similarly another participant struggles to comprehend the meaning of ‘mental effort or concentration’, viewing breathing as automatic, a natural bodily response and so beyond concentration. In wrestling with this question, he explores whether ‘mental effort’ has something to do with mental health:

Well, what’s mental effort? Means I’ve got a hard job to breathe? *I’m quite normal of breathing, er, and I ain’t got to concentrate on breathing,* if you get what I’m on about. Concentrate, I suppose, is, you’ve got to think, “Oh, I’ve got to breathe, I’ve got to breathe,” or something like that. I say, no, I don’t think that applies to me…“Breathing requires mental effort or concentration”…that confuses me…what’s mental in breathing? Oh, you’ve got to breathe. Breathe! So if you’re saying that then, you’re up here [pointing to head]…your mental health is telling you, “Oh, you’ve got to breathe.” Well, I don’t need that because I am breathing. And I don’t need the concentration. I don’t know [laughs]—who puts these questions?…I mean if you’re exhausted or out of breath, breathing becomes automatic, you can’t stop it, can you?…I mean if I’m out of breath it’s just natural [makes panting sound]. *It becomes automatic…Um, you might think about it but, um, it doesn’t require any mental effort to do it, concentration*. I mean if I am breathless I’m puffing like billy-o… (BE-BA-R)

It is possible to see how confused the participant is by the question in part because he views and experiences the breath (and even breathlessness) as an automatic bodily process, “I’m quite normal of breathing,” which does not require conscious mental thought processes: “your mental health is telling you, ‘Oh, you’ve got to breathe’. Well, I don’t need that because I am breathing.” He separates out concentration and mental effort from ‘thinking about it’: “you might think about it but it doesn’t require any mental effort to do it, concentration.”

## Discussion

### Anthropology and cognitive interviewing

In the data presented we have explored the multiple ways participants wrestled with the meaning of the words and phrases used to describe breathless sensations in four items on the MDP questionnaire. Three strategies people may use to cope with problematic questionnaire items have been put forward by Galasinski: reformulate, recontextualise and contest.58 Reformulate refers to answering different questions from those posed. Recontextualise refers to drawing on contexts that render questions non-sensical. Contest refers to challenging assumptions underlying the scale, as irrelevant, insensitive or distressing. We can see good examples of all three strategies being used in relation to the MDP.

Margaret Lock’s concept of ‘local biology’ may be a useful way to explain *why* participants feel the need to reformulate, recontextualise and contest questionnaire items on respiratory questionnaires.59 Lock’s notion of local biologies was an early precursor to biological medical anthropology in that her theory was that physical biology is not universal. Our biology is shaped, in a very real sense (not just symbolically), by the cultural-ecological-geographical contexts in which we live and (linking to epigenetics) in which our ancestors lived. Local biologies are linked with both the experience and the interpretation of sensations. For example, ‘bodily noise’ in the form of multiple physical ailments (associated with local biologies of social deprivation in Denmark) meant that symptoms were normalised and sensitivity to sensations was reduced.60, 61 This is similar to a work in the UK suggesting breathlessness is normalised within communities from lower socioeconomic regions, where smoking is prevalent.62

The local biology in this study was shaped by smoking prevalence rather than social-intergenerational deprivation (as in the Danish study), with participants representing a range of professionals, including social workers, counsellors, linguists, retired army officers, chefs, engineers and palliative carers. Yet, when a woman turns up breathless to a group (attended by AM) and is soothed by the comment ‘old age never arrives alone’, her breathlessness is normalised as a natural part of ageing. Breathlessness in these socioeconomic cultures is not audible; the bodily noise is disregarded as ‘symptomatic’ of respiratory disease. For example in our data, for years before receiving a diagnosis of COPD, one participant who experienced breathlessness described:

every time I complained about coughs, and, you know, severe coughs, the doctors would not really take any notice…then one doctor said to me, “We’ve got a spirometer now, you could try that if you wanted to.” But it was so casual and it sounded as though I was being a bit of a nuisance, (laughs) so I left it. (BE_C-B)

In this example, it is not that those living with breathlessness do not have symptoms and understandings of their meanings, it is that their interpretations are met by a lack of interest in sensations of breathlessness by clinicians, which comes to be internalised by those experiencing them—the noisy body is not listened to.63 In Fricker’s conceptualisation of hermeneutical epistemic injustice, marginalised social groups are subjected to epistemic harms due to a silence or gap in knowledge.64 Yet the ‘local biologies’ of breathlessness can be understood more in the framework suggested by Dotson, which identified the way that power affects the extent to which the dominant discourse considers ‘alternative epistemologies, counter mythologies, and hidden transcripts that exist in hermeneutically marginalised communities *among themselves*’.65 It is this framework that Braun and Kopinski draw on to explain the normalisation of suffering among communities of mine workers: ‘publicly-funded science privileges certain accounts of disease and excludes other accounts, such as those of the asbestos workers on the mines’.66 Similarly, as McGuire explains in the context of the struggle to define levels of respiratory disability between 1939 and 1945, the miners involved had nuanced and sophisticated awareness of their breathlessness, but the Medical Research Council were unable to standardise this type of knowledge into the categorisation systems required for objectivity and compensation calculability.67

An anthropological critique of the quantitative assumptions underlying measures such as the MDP is needed not least because ‘whilst instruments are ideally supposed to transcend biography, culture and history, they (cannot but) fail to do so and this needs to be evaluated’68. The difficulty inherent to using instruments to create objective measures has long historical precedent and has been observed by scholars in specific cases such as Alzheimer’s disease69 and depression,70 as well as more broadly in relation to psychiatry,71 disability72 and medicine.73 In the case of breathlessness, the history of its measurement may shed light on this recurring disjunct between objective and subjective measures. Williams and Carel have argued that the privileging of the physiological symptoms of breathlessness has resulted from the medical model’s attempts to define breathlessness in ways that fail to account for the lived experience of the patient.74 However, the drive to translate breathlessness into quantifiable and scalable measures is inter-related with historical links between respiratory disease, industry and compensation.75 The administrative processes involved in compensation for lung disease necessitated defining strict levels of illness, which could ideally be expressed numerically. However, as explored in this article, and in the work of Carel and Oxley and Macnaughton, there is a considerable disjunct between breathlessness as it is experienced and the objective correlate.76, 77

The idea that this could lead to epistemic injustice is reinforced by consideration of the historical fight for recognition and compensation of ‘miners’ lung’. Braun has already indicated that the spirometer was a key factor in this struggle, as the device had accepted epistemic authority for both the miners and the medics.78 The case of ‘miners’ lung’ is a key example of how medical testing can conflict with the experiential correlate. This can be seen both in the USA and in the UK.79, 80 For example, by examining the Medical Research Council’s measurement of lung function in British miners from 1936 to 1945, we can see that the threshold for normal lung function was taken from a baseline measurement of other miners, rather than a normal comparison group. That is, healthy lung capacity for the purpose of assessing respiratory disability was what was normal for miners, meaning those who *felt* their respiratory level was diminished could be dismissed as healthy based on apparently objective scientific measurements.81 Trusting in measurements over the testimony of the measured can lead to what we might term ‘mechanical epistemic injustice’. Moreover, the kind of mechanical epistemic injustice we see in the case of the miners can arise because, as Haslanger explains, the failure to recognise the structures that give rise to the regularities leads us to attribute the regularities to something intrinsic to the agents.82 The miners’ claims of breathlessness were dismissed by the superior objective evidence from X-rays and spirometric measurements—the normal baseline for spirometric data had been configured not to healthy controls, but to the miners themselves. This attitude reflected the prevalent ‘local biologies’ of coalminers, who would often continue to work while disabled in a context in which disability was not statistically abnormal.83

Nuanced understanding of the history of breathlessness measurement shows how much is at stake when interpreting sensations. The internal nature of breathlessness poses a communication problem between the clinician and the patient ‘because there is no external reference that can be measured’,84 *and* because local biologies make the experience of breathlessness socially inaudible. So the problem is, if we know the MDP is a validated and reliable measure of participants’ condition and recovery, ‘How do we reconcile psychometric credibility based upon quantitative measures of reliability and validity with qualitative analysis that potentially raises questions about the utility of a measure?’85

In other words, are the quests for meaning and for measurement incommensurable research objectives? Beyond our own work, there are multiple examples from transcultural psychiatry suggesting the two things are often incommensurable.86 For example, a research team using a Spanish translation of the Edinburgh Postnatal Depression Scale with women in Mexico found the inclusion of the word ‘desgraciada’ led women to rate themselves as not having symptoms, not because they did not feel sadness, but because the term ‘desgraciada’ used to describe sadness was offensive, with the meaning in some regions of ‘wretched, unlucky, child of a prostitute’. The meaning of the item on this questionnaire had been lost in translation, leading the results to be skewed and the research team to urge others to investigate possible differences in interpretation of words or phrases for ‘other significant unaccounted for intraethnic differences’ in meaning.87

Another informative example of the tensions between meaning and measurement is the unprecedented inclusion of distinctly non-Western health beliefs for a number of items on an anxiety and depression scale. Phan, Steel and Silove describe the powerful influence of cosmological beliefs on cultural meanings of low mood, such as the inclusion of the item, Ca’m thây không có niêm tin o’’ tu’o’ng lai? (Lost hope in fate). The influence of traditional Chinese health explanations is particularly evident in the description of a number of symptoms, such as one item that was added to the somatic distress scale, Bi. na.ng mat, nhú’c mat, nhìn thây dom dóm, which translates to ‘had heavy eyes as if you were seeing rainbows’, which appears to have no apparent equivalent experience among Anglo-European populations.88 This work illustrates how much may be lost in translation if tools of measurement are not adapted to include the meanings embedded within cultural contexts and local biologies.

One possible explanation for the mismatch between ‘meaning making’ and ‘measurement is that:

questionnaires work within the parameters of dominant discourse of clinical (settings) and so successfully measure something because it corresponds with the rules of what constitutes such measurement. And while it might identify (clinical diagnostic labels) it is unlikely to pin down individual experiences (of breathlessness).89

As Hinton reminds us, sensory meaning is never simply a question of physiology; it is always mediated by culture, in the sense of the ways of life, language, ritual practices, beliefs and aesthetics of a group, community or society.90

In the clinical context the idea of symptom assessment and consequent diagnosis depends on the idea of accuracy.91 The assumption is that a participant’s sensory experience of a symptom is directly related to a measurable physiological abnormality. Van den Bergh *et al* go so far as to say that this ‘accuracy assumption’ represents a ‘fundamental implicit contract among the patient, the physician, and the healthcare system’.92 The problem is that this assumption holds true only when the relationship between physiological stimulus and perceived symptom is a simple one, such as that between cardiac arrhythmias and palpitations. In the context of chronic, multisystem conditions, with complex sets of stimuli across a range of bodily systems, not to mention the influence of the symptom in the context of a long life, this simple relationship starts to break down. If, then, we recognise that the ‘accuracy assumption’ does not necessarily hold true we may need to start asking different questions to measure the significance of a symptom like breathlessness. For example, if instead of measuring the relationship between measured pulmonary function and frequency of hospital admissions we ask a different question such as ‘What is the relationship between breathlessness perception and risk of death’, we may get a more meaningful answer. By asking this question, Nishimura *et al* showed that breathlessness perception was a better predictor of mortality than objectively measured lung function.93

### Study limitations

A limitation of the work presented here is that it represents only the experiences of those attending the Breathe Easy groups. The social demographics of the Breathe Easy groups AM was in contact with were all white despite the surrounding localities being ethnically diverse, meaning one criticism of the MDP—that it has been developed with a homogeneous white population—has been inadvertently reproduced in this study. The reasons why the Breathe Easy groups do not attract more members from the wider black and ethnic minority groups within their local community is a research question for future work. Our future work on the problematic nature of breathlessness measurement should move beyond the Breathe Easy communities in order to explore these same issues with a more ethnically diverse population.

## Conclusion

Part of a medical humanities and critical anthropology approach to exploring the accuracy assumption in the measurement of breathlessness is to understand how it is that clinical science has arrived at the descriptors that the respondents in the cognitive interview study are offered on the MDP questionnaire. The presentation in this article of the multiple examples of reinterpretation, miscomprehension of questionnaire items and the language used is, therefore, symptomatic of the limitations of the MDP. Underlying causes for this symptomology are as follows: Absence of a more interdisciplinary approach to developing measures such as the MDP results in missed opportunities to include conversations with medical humanities scholars, anthropologists, neuroscientists and historians in the early stages of developing a new respiratory measure. Similarly, the absence of ‘experts by experience’ contributes in a meaningful way to the design and testing of the measure, in particular its resonance with a lived experience. Lastly is the absence of a robust ‘ease of use’ testing and face validity ‘testing’, what we have referred to here as ‘interpretative measurement error’.

Jo Winning asks: ‘What is a body? What are its boundaries and contours? How do we come to know the body through the senses’.94 In this paper we have explored the variability in participant readings of clinically described ‘respiratory symptoms and sensations’, revealing a vocabulary of descriptors of breathlessness that is more nuanced and representative of the lived experience. We have related these local vocabularies to a historically influenced account of mechanistic epistemic injustice. It is, Winning argues, one of the key tasks of critical medical humanities to establish a transdisciplinary dialogue across the domains of clinical medicine and critical thought, offering clinical medicine new terms and concepts to strengthen its ongoing dealings with the human body. As Winning puts it: ‘transdisciplinary explorations across the domains of the clinic and culture begin to show ways in which new sensate and sensitive vocabularies might be brought into clinical practice’.95

In the Life of Breath project, we have tended to prioritise the theme of ‘invisibility’ in our explorations of breathlessness. Here we have put forward ideas of breathlessness in terms of cultural audibility—exploring which localities and contexts may silence the experience of sensations, while other socioeconomic contexts make bodies, and breathless symptoms, culturally audible, even loud. When participants from within these regions of low socioeconomic growth are invited to think aloud their thoughts in response to the MDP, the body speaks up, hence the title of our article. Our work suggests allowing ‘the body to speak’ and acknowledging the various cultures and local biologies that make some bodies noisier than others will be an important part of that conversational process with clinical medicine. Situating this conversation within a historical account of previous examples of mechanistic epistemic injustice is also vital if we are to move towards a more reconciliatory meeting of competing (local) biologies.

### Note on terminology

We use ‘disabled’ rather than ‘people with disabilities’ in line with practices from disability studies intended to highlight the ways in which we are disabled by (eg, people, practices, workplaces) and so as not to perpetuate the idea that the word is a pejorative.

## Data Availability

Data are available upon reasonable request. Copies of the topic guides and transcripts of full interviews are available from the lead author on request.

## Notes

87. Janet M Wojcicki and Jillian Geissler (2013), “The Spanish Translation of the Edinburgh Postnatal Depression Scale and the Use of the Word ‘Desgraciada’,” *Transcultural Psychiatry* 50, no. 1: 153.

88. Tuong Phan, Zachary Steel, and Derrick Silove (2004), “An Ethnographically Derived Measure of Anxiety, Depression and Somatization: The Phan Vietnamese Psychiatric Scale,” *Transcultural Psychiatry* 41, no. 2: 200–32.

89. Galasiński, “Constructions of the Self,” 259, 515–33.

90. Hinton, Howes and Kirmayer, “Toward a Medical Anthropology of Sensations,” 143.

91. Omer Van den Bergh, Nadia Zacharioudakis, and Sibylle Petersen (2018), “Interoception, Categorization, and Symptom Perception,” in *The Interoceptive Mind: From Homeostasis to Awareness,* eds. Manos Tsakiris and Helena de Preester (Oxford: Oxford University Press), 212.

92. Van den Bergh, Zacharioudakis and Petersen, “Interoception, Categorization, and Symptom Perception,“213.

93. Koichi Nishimura et al. (2002), “Dyspnea Is a Better Predictor of 5-Year Survival Than Airway Obstruction in Patients With COPD,” *Chest* 121, no. 5: 1434–40.

94. Joanne Winning (2016), “Afterword: The Body and the Senses,” in *The Edinburgh Companion to the Critical Medical Humanities,* eds. A. Whitehead *et al*. (Edinburgh University Press), 325.

95. Winning, “Afterword,” 325.

96. Robert Banzett et al. (2015), Multidimensional dyspnea profile: aninstrument for clinical and laboratory research.” *European Respiratory Journal* 45, no. 6.

1. Marianne Rosendal et al. (2013), “Multiple Perspectives on Symptom Interpretation in Primary Care Research,” *BMC Family Practice* 14, no. 1: 5, 167.

2. Robert W Lansing, Richard H Gracely, and Robert B Banzett (2009), “The Multiple Dimensions of Dyspnea: Review and Hypotheses,” *Respiratory Physiology & Neurobiology* 167, no. 1: 53–60.

3. David Howes (2005), *Empire of the Senses* (Oxford: Berg Publishers).

4. Devon E Hinton, David Howes, and Laurence J Kirmayer (2008), “Toward a Medical Anthropology of Sensations: Definitions and Research Agenda,” *Transcultural Psychiatry* 45, no. 2: 142–62.

5. David Howes (2019), Multisensory Anthropology.” Annual Review of Anthropology 48, no. 1: 17–28.

6. David Howes (2012), “The Extended Sensorium: Introduction to the Sensory and Social Thought of François Laplantine,” in *The Life of the Senses: Introduction to a Modal Anthropology* (London: Bloomsbury Academic), vii–xiv.

7. Howes, “The Extended Sensorium,” vii–xiv.

8. Françcois Laplantine (2015), *The Life of the Senses: Introduction to a Modal Anthropology* (London: Bloomsbury Academic), 83.

9. Hinton, Howes, and Kirmayer, “Toward a Medical Anthropology of Sensations,” 142–62.

10. Megan Wainwright (2017), “Sensing the Airs: The Cultural Context for Breathing and Breathlessness in Uruguay,” *Medical Anthropology* 36, no. 4: 332–47.

11. Brian Lande (2007), “Breathing Like a Soldier: Culture Incarnate,” *The Sociological Review* 55 (Suppl. 1): 95–108.

12. Megan Wainwright (2018), “Imaging and Imagining Chronic Obstructive Pulmonary Disease (COPD): Uruguayans Draw Their Lungs,” *Disability and Rehabilitation* 40, no. 26: 3094–103.

13. Robert B Banzett and Shakeeb H Moosavi (2017), “Measuring Dyspnoea: New Multidimensional Instruments to Match Our 21st Century Understanding,” *European Respiratory Journal* 49, no. 3: 1–6, 1602473.

14. Robert Banzett *et al*. (2015), “Multidimensional Dyspnea Profile: An Instrument for Clinical and Laboratory Research,” *European Respiratory Journal* 45, no. 6: 1681–91.

15. C Bausewein et al. (2007), “Measurement of Breathlessness in Advanced Disease: A Systematic Review,” *Respiratory Medicine* 101, no. 3: 399–410.

16. Capucine Morélot-Panzini et al. (2016), “Real-Life Assessment of the Multidimensional Nature of Dyspnoea in COPD Outpatients,” *European Respiratory Journal* 47, no. 6: 1668–79.

17. Magnus Ekström and Josefin Sundh (2016), “Swedish Translation and Linguistic Validation of the Multidimensional Dyspnoea Profile,” *European Clinical Respiratory Journal* 3, no. 1: 32665.

18. Leticia F Belo et al. (2019), “A Breath of Fresh Air: Validity and Reliability of a Portuguese Version of the Multidimensional Dyspnea Profile for Patients With COPD,” *PloS One* 14, no. 4: e0215544.

19. Marie T Williams et al. (2017), “Comparison of the Dyspnoea-12 and multidimensional dyspnoea profile in people with COPD.” European Respiratory Journal 49, no. 3.

20. Paula M Meek et al. (2012), “Reliability and Validity of the Multidimensional Dyspnea Profile.” Chest 141, no. 6.

21. Robert B. Banzett, Sarah H. Pedersen, Richard M. Schwartzstein, and Robert W. Lansing (2008), “The Affective Dimension of Laboratory Dyspnea: Air Hunger Is More Unpleasant Than Work/Effort,” *American Journal of Respiratory and Critical Care Medicine* 177, no. 12: 1384–90.

22. Mark B Parshall (2002), “Psychometric Characteristics of Dyspnea Descriptor Ratings in Emergency Department Patients With Exacerbated Chronic Obstructive Pulmonary Disease,” *Research in Nursing & Health* 25, no. 5: 331–44.

23. Mark W Elliott et al. (1991), “The Language of Breathlessness,” *American Review of Respiratory Disease* 144, no. 23: 826–32.

24. P M Simon et al. (1990), “Distinguishable Types of Dyspnea in Patients With Shortness of Breath,” *American Review of Respiratory Disease* 142, no. 5: 1009–14.

25. Parshall, “Psychometric Characteristics of Dyspnea Descriptor Ratings,” 335.

26. Personal communication cited in Robert Banzett and Shakeeb Moosavi, “Measuring Dyspnoea,” 3, 1602473.

27. Lansing, Gracely and Banzett, “The Multiple Dimensions of Dyspnea,” 53–60.

28. Parshall, “Psychometric Characteristics of Dyspnea Descriptor Ratings,” 331–44.

29. G E Hardie et al. (2000), “Ethnic Differences: Word Descriptors Used by African-American and White Asthma Patients during Induced Bronchoconstriction,” *Chest* 117, no. 4: 935–43.

30. Steven Epstein (2004), “Bodily Differences and Collective Identities: The Politics of Gender and Race in Biomedical Research in the United States,” *Body & Society* 10, no. 2–3: 183–203.

31. Heather Munro Prescott (2002), “Using the Student Body: College and University Students as Research Subjects in the United States during the Twentieth Century,” *Journal of the History of Medicine and Allied Sciences* 57, no. 1: 3–38.

32. The expectation that we should include women, children, the elderly and ethnic minorities in clinical trials was only legally stipulated in the 1990s, the result of activism by concerned groups in the 1980s. And yet the use of the average white male as standard research subject was inspired by a desire to protect ‘vulnerable groups’ from risky research after the Second World War rather than simple discrimination. See Steven Epstein (2007), *Inclusion: The Politics of Difference in Medical Research* (Chicago: Chicago University Press), 30–3.

33. Anja Hayen, Mari Herigstad, and Kyle T S Pattinson (2013), “Understanding Dyspnea as a Complex Individual Experience,” *Maturitas* 76, no. 1: 46.

34. Hayen, Herigstad and Pattinson, “Understanding Dyspnea,” 46.

35. Lansing, Gracely and Banzett, “The Multiple Dimensions of Dyspnea,” 57.

36. Jane Macnaughton and Havi Carel (2016), “Breathing and Breathlessness in Clinic and Culture: Using Critical Medical Humanities to Bridge an Epistemic Gap,” in *The Edinburgh Companion to the Critical Medical Humanities,* eds. A. Whitehead *et al*. (Edinburgh University Press).

37. Miranda Fricker (2007), *Epistemic Injustice: Power and the Ethics of Knowing* (Oxford: Oxford University Press).

38. Havi Carel and Ian James Kidd (2014), “Epistemic Injustice in Healthcare: A Philosophical Analysis,” *Medicine, Health Care and Philosophy* 17, no. 4: 529–40.

39. Newspaper clippings: “’ Questionnaire,” *South Wales Echo,* January 16, 1950, and “Wives Ban ‘Means Test’ in Pit Disease Check,” *South Wales Echo*, undated, both accessed at South Wales Miners Library (SWML).

40. The Life of Breath study is a 5-year interdisciplinary project led by teams from Bristol and Durham universities and funded by the Wellcome Trust (grant numbers: 103339/Z/13/Z; 103340/Z/13/Z) to explore phenomenological, cultural, historical and clinical understandings of breathlessness.

41. Mari Herigstad et al. (2011), “Dyspnoea and the Brain,” *Respiratory Medicine* 105, no. 6: 809–17.

42. “Pulmonary Rehabilitation: What Is Pulmonary Rehabilitation (PR)?” Information on NHS England website, accessed September 2020, https://www.england.nhs.uk/ourwork/clinical-policy/respiratory-disease/pulmonary-rehabilitation /

43. Alice Martin et al. (2012), “Effect of Ethnicity on the Prevalence, Severity, and Management of COPD in General Practice,” *British Journal of General Practice* 62, no. 595: e76–e81.

44. Gerard J Criner et al. (2020), “Racial Disparities: Another Important Barrier to Pulmonary Rehabilitation Participation in Patients With COPD Posthospitalization,” *Chest* 157, no. 5: 1070–2.

45. Dara Culhane and Denielle Elliott (2016), *A Different Kind of Ethnography: Imaginative Practices and Creative Methodologies* (Toronto: University of Toronto Press).

46. Elspeth Penny and Alice Malpass (2019), “Dear Breath: Using Story Structure to Understand the Value of Letter Writing for Those Living With Breathlessness – A Qualitative Study,” *Arts and Health* 15, no. 1: 1–15 (available as preprint).

47. Alice Malpass and Elspeth Penny (2019), “Invisible Breath,” *Storytelling, Self, Society* 15, no. 1: 43–70.

48. William Viney, Felicity Callard, and Angela Woods (2015), “Critical Medical Humanities: Embracing Entanglement, Taking Risks,” *Medical Humanities* 41, no. 1: 2–7.

49. Edmund Ronald Leach (1967), “An Anthropologist’s Reflections on a Social Survey,” in *Anthropologists in the Field*, eds. D.G. Jongmans and P.C.W. Gutkind (Assen: Van Gorcum), 81.

50. Norbert Schwarz (2007a), “Cognitive Aspects of Survey Methodology,” *Applied Cognitive Psychology* 21, no. 2: 278.

51. Jenny Hockey and Martin Forsey (2012), “Ethnography Is Not Participant Observation: Reflections on the Interview as Participatory Qualitative Research,” in *The Interview: An Ethnographic Approach,* ed. Jonathan Skinner (London: Bloomsbury Publishing), 69–87.

52. Judith Okely (2011), “Retrospective Reading of Fieldnotes. Living on Gypsy Camps,” *Behemoth* 4, no. 1: 18–42.

53. Okely, “Retrospective Reading of Fieldnotes,” 20.

54. Joanna Smith and Jill Firth (2011), “Qualitative Data Analysis: The Framework Approach,” *Nurse Researcher* 18, no. 2: 52–62.

55. MDP, online supplement, 3 from Meek, Paula M., Robert Banzett, Mark B. Parsall, Richard H. Gracely, Richard M. Schwartzstein, and Robert Lansing. "Reliability and validity of the multidimensional dyspnea profile." *Chest* 141, no. 6 (2012): 1546-1553.

56. M L Moy et al. (1998), “Language of Dyspnea in Assessment of Patients With Acute Asthma Treated With Nebulized Albuterol,” *American Journal of Respiratory and Critical Care Medicine* 158, no. 3: 749–53.

57. Mark B Parshall (2002), “Psychometric Characteristics of Dyspnea,” 331–44.

58. Dariusz Galasiński (2008), “Constructions of the Self in Interaction With the Beck Depression Inventory,” *Health: An Interdisciplinary Journal for the Social Study of Health, Illness and Medicine* 12, no. 4: 515–33.

59. Margaret Lock (2001), “The Tempering of Medical Anthropology: Troubling Natural Categories,” *Medical Anthropology Quarterly* 15, no. 4: 478–92.

60. Lock, “Tempering of Medical Anthropology,” 483.

61. Camilla Hoffmann Merrild (2017), “Noisy Lives, Noisy Bodies: Exploring the Sensorial Embodiment of Class,” *Anthropology in Action* 24, no. 1: 13–19.

62. Rebecca Oxley and Jane Macnaughton (2016), “Inspiring Change: Humanities and Social Science Insights Into the Experience and Management of Breathlessness,” *Current Opinion in Supportive and Palliative Care* 10, no. 3: 256.

63. Lenore Manderson and Gabrielle Rose (2000) “More Than a Breath of Difference: Competing Paradigms of Asthma.” Anthropology & Medicine 7, no. 3

64. Miranda Fricker (2007), *Epistemic Injustic*e.

65. Kristie Dotson (2012), “A Cautionary Tale: On Limiting Epistemic Oppression,” *Frontiers: A Journal of Women Studies* 33, no. 1: 24–47.

66. Lundy Braun and Hannah Kopinski (2018), “Causal Understandings: Controversy, Social Context, and Mesothelioma Research,” *BioSocieties* 13, no. 3: 557–79.

67. Coreen McGuire (2019), “‘X-Rays Don’t Tell Lies’: The Medical Research Council and the Measurement of Respiratory Disability, 1936-1945,” *The British Journal for the History of Science* 52, no. 3: 447–65.

68. Dariusz Galasiński (2008), “Constructions of the Self,” 523.

69. Duncan Wilson (2017), “Calculable People? Standardising Assessment Guidelines for Alzheimer’s Disease in 1980s Britain,” *Medical History* 61, no. 4: 500–24.

70. Michael Worboys (2013a), “The Hamilton Rating Scale for Depression: The Making of a ‘Gold Standard’ and the Unmaking of a Chronic Illness, 1960-1980,” *Chronic Illness* 9, no. 3: 202–19.

71. Martyn Pickersgill (2012), “What Is Psychiatry? Co-Producing Complexity in Mental Health,” *Social Theory & Health* 10, no. 4: 328–47.

72. Matthew Kohrman (2003), “Why Am I Not Disabled? Making State Subjects, Making Statistics in Post--Mao China,” *Medical Anthropology Quarterly* 17, no. 1: 5–24.

73. Stefan Timmermans and Marc Berg (2010), *The Gold Standard: The Challenge of Evidence-Based Medicine and Standardization in Health Care* (Philadelphia: Temple University Press).

74. Tina Williams and Havi Carel (2018), “Breathlessness: From Bodily Symptom to Existential Experience,” in *Existential Medicine* (London: Rowman & Littlefield International).

75. Theodore M Porter (2003), “FOCUS ARTICLE: Measurement, Objectivity, and Trust.” Measurement: Interdisciplinary Research and Perspectives 1, no. 4.

76. Havi Carel (2018), “Breathlessness: The Rift between Objective Measurement and Subjective Experience,” *The Lancet Respiratory Medicine* 6, no. 5: 332–3.

77. Rebecca Oxley and Jane Macnaughton (2016), “Inspiring Change,” 256.

78. Lundy Braun (2014), *Breathing Race Into the Machine: The Surprising Career of the Spirometer From Plantation to Genetics* (Minneapolis: University of Minnesota Press).

79. B E Smith and Barbara Ellen Smith (1981), “Black Lung: The Social Production of Disease,” *International Journal of Health Services* 11, no. 3: 343–59.

80. McGuire, “X-Rays Don’t Tell Lies,” 447–65.

81. Ibid.

82. Sally Haslanger (2017), “Objectivity, Epistemic Objectification, and Oppression,” in *The Routledge Handbook of Epistemic Injustice,* eds. Ian James Kidd, Jose Mdina and Gaile Pohlhaus, Jr. (London: Routledge), 279–90.

83. David M Turner and Daniel Blackie (2018), *Disability in the Industrial Revolution: Physical Impairment in British Coalmining, 1780–1880* (Manchester: Manchester University Press).

84. Lansing, Gracely and Banzett, “The Multiple Dimensions of Dyspnea,” 53–60.

85. Duriusz Galasiński and Olga Kozłowska (2010), “Questionnaires and Lived Experience: Strategies of Coping With the Quantitative Frame,” *Qualitative Inquiry* 16, no. 4: 281.

86. M Cameron Hay (2008). “Reading Sensations: Understanding the Process of Distinguishing 'Fine' From 'Sick'.” Transcultural Psychiatry 45, no. 2 (2008): 198–229.
